# Impact of a probiotic product on bowel habits and microbial profile in participants with functional constipation: A randomized controlled trial

**DOI:** 10.1111/1751-2980.12797

**Published:** 2019-08-01

**Authors:** Christopher J. Martoni, Malkanthi Evans, Cheryl‐Emiliane T. Chow, Luisa S. Chan, Gregory Leyer

**Affiliations:** ^1^ UAS Laboratories Windsor Wisconsin USA; ^2^ KGK Science London Ontario Canada; ^3^ Second Genome South San Francisco California USA

**Keywords:** bowel habit, functional constipation, microbiome, probiotic, randomized controlled trial

## Abstract

**Objective:**

To investigate the clinical efficacy of a multi‐strain probiotic product on bowel habits and microbial profile in participants with functional constipation.

**Methods:**

This was a randomized, double‐blind, placebo‐controlled and parallel‐arm study. Altogether 94 otherwise healthy adults aged 18 to 65 years with symptoms of functional constipation were randomized as part of the intention‐to‐treat population. The participants received a placebo or the probiotic product (1.5 × 10^10^ CFU/day), consisting of *Lactobacillus acidophilus* DDS‐1, *Bifidobacterium animalis* subsp. *lactis* UABla‐12, *Bifidobacterium longum* UABl‐14 and *Bifidobacterium bifidum* UABb‐10 over 4 weeks. Outcomes included the patient assessment of constipation‐symptom (PAC‐SYM) questionnaire, stool frequency and consistency, and microbial profile.

**Results:**

There were no significant between‐group differences in the PAC‐SYM score, despite significant within‐group differences (*P* < 0.001) over the study period. The probiotic group showed a faster normalization of stool frequency and consistency, with most participants achieving a normalized profile after 1 week. Fecal samples of the probiotic group exhibited higher relative abundance of Ruminococcaceae (*P* = 0.0047), including the *Ruminococcus* genus, and lower relative abundance of Erysipelotrichaceae (*P* = 0.0172) at end‐point compared with baseline. Placebo group samples showed similar abundance profiles over the study, with the exception of Clostridiaceae, which was lower at the study end‐point (*P* = 0.0033). Among treated participants, all four probiotic strains were significantly more abundant after the intervention.

**Conclusions:**

No significant differences were observed in symptomology, with both groups showing a more than 20% improvement. However, the probiotic helped modulate bowel function earlier than the placebo, with a corresponding shift to a more fibrolytic microbiota.

## 1 INTRODUCTION

Functional gastrointestinal disorders, including functional constipation, are among the most frequently observed conditions in clinical practice.^1^ Functional constipation is symptom‐based, non‐organic in origin and commonly diagnosed by Rome IV diagnostic criteria.^2^ The disorder has no known structural abnormalities, infectious or metabolic causes.^3^ It has an estimated prevalence of 14% (95% CI 12‐17%) in adults,^4^ representing a significant health care burden. Constipation and digestive symptoms associated with discomfort result in absenteeism from work and lost productivity as well as reduced quality of life and increased medical costs.^5^ Furthermore, patients' perception of their symptoms may be amplified by physiological, intrapsychic, and sociocultural factors that influence their daily life activities.^1^, ^5^

The etiology and pathophysiology of functional constipation are most likely multifactorial. Previous studies have found chronic constipation to be related to dysbiosis^6^, ^7^, ^8^, ^9^ and such alterations have been suggested as a possible pathophysiologic mechanism.^10^ Prolonged gastrointestinal transit time may also lead to dysbiosis, which can in turn affect motility as well as immune and barrier function.^11^, ^12^ Nevertheless, research into the relationship between constipation and a dysbiotic microbiome is in its infancy.

The clinical management of functional constipation remains challenging. Current management options include diet and lifestyle changes, as well as the use of bulking agents, stool softeners, osmotic and stimulant laxatives, and prescription drugs.^3^, ^13^ However, many such products have limitations due to a lack of efficacy, inconsistent symptom response or safety concerns.^14^ Lately, probiotics have been adopted as an adjunct approach to normalize intestinal transit time and alleviate symptoms.^15^

Probiotics are defined as “live microorganisms that, when administered in adequate amounts, confer a health benefit to the host”.^16^ The safety profile of probiotics and fermented foods has been well documented in the general population; however, recent safety determination guidance for strain assessments^17^ and commentaries for probiotic use in at‐risk populations^18^ have been published. A recent systematic review and meta‐analysis concluded that probiotics might improve whole gut transit time and stool frequency profile in adults with functional constipation, albeit with high heterogeneity across studies.^15^ In a separate meta‐analysis, probiotic supplementation was found to decrease intestinal transit time, with statistically superior effects observed in adults with constipation or of advanced age.^19^ However, the ability of probiotics to improve intestinal transit times is generally believed to be strain‐specific^20^ and improvements observed in transit time have not necessarily been associated with improved symptomology. As such, more studies are warranted examining the utility of probiotics in relieving functional constipation.^20^ Also, despite evidence of an association of functional constipation with dysbiosis, few randomized controlled trials with probiotics have looked at microbial profiling and symptom outcomes simultaneously.

A probiotic blend, consisting of *Lactobacillus acidophilus* DDS‐1*, Bifidobacterium animalis* subsp. *lactis* UABla‐12*, B. longum* UABl‐14 and *B. bifidum* UABb‐10, was previously shown*,* in a pilot clinical trial, to improve symptomology in participants with irritable bowel syndrome.^21^ The current randomized controlled trial aimed to evaluate the clinical efficacy of this multi‐strain probiotic on bowel habits, symptomology, microbial profiling and strain recovery in otherwise healthy participants with functional constipation.

## 2 PARTICIPANTS AND METHODS

### 2.1 Study population

This study was conducted in accordance with the ethical principles that have their origins in the Declaration of Helsinki and subsequent amendments. Notice of authorization for the study (File no. 208099) was granted by the Natural and Non‐Prescription Health Products Directorate (Ottawa, Ontario, Canada). An institutional review board (IRB Services, Aurora, Ontario, Canada) provided unconditional approval for the study (IRB reference no. Pro00011887). The clinical trial was registered on http://clinicaltrials.gov under registration number NCT02418507, and was conducted according to the CONSORT 2010 Statement.

Adults aged 18 to 65 years with symptoms of functional constipation were recruited at KGK Science (London, Ontario, Canada). The included participants met the requirements of Rome III criteria for functional constipation based on self‐reporting over the past 3 months, with symptom onset beginning within the previous 6 months. Additionally, participants were required to have an average stool type of <3 on the Bristol stool scale (BSS), as assessed over a 2‐week run‐in period and agreed to maintain their current level of physical activity throughout the trial period. Exclusion criteria included women who were pregnant, breastfeeding, or intended to get pregnant, those with type I or type II diabetes, cancer, neurological disorders, immunocompromised conditions, major diseases of the cardiovascular, renal, hepatic, gastrointestinal, pulmonary or endocrine systems, a history of gastrointestinal complications (such as inflammatory bowel disease and ulcers) or abdominal surgery, a history of heavy drinking or an allergy or sensitivity to the test product ingredient. The use of antibiotics, probiotics, fiber supplements or prebiotic fiber and enriched foods were prohibited within 4 weeks prior to screening and during the trial. All participants provided their voluntary, written, informed consent prior to their inclusion.

### 2.2 Study design

This was a prospective, randomized, placebo‐controlled, double‐blind and parallel‐arm study. Investigational visits took place at screening and at baseline (d 0), mid‐point (d 15 ± 3) and end‐point (d 29 ± 3) of the 4‐week intervention period. At screening, the participants’ complete medical history, concomitant therapies and inclusion criteria were reviewed, their characteristics and vital sign measures were recorded and fasted blood was collected for hematological and biochemical assessments.

Participants meeting the screening criteria entered a 2‐week run‐in period, which involved the completion of a daily bowel habit diary, 3‐day food records and a physical activity questionnaire. Eligibility was confirmed at baseline, and included a review of their inclusion criteria, a physical examination, vital sign assessments, weight and body mass index (BMI) measurements, completion of patient assessment of constipation ‐ symptom (PAC‐SYM) and patient assessment of constipation ‐ quality of life (PAC‐QoL) questionnaires, and, if applicable, a urine pregnancy test. The study diaries, including the records of daily bowel habits, food and physical activity questionnaires were collected. Eligible participants were randomized to receive placebo or probiotic capsules (1:1). All study personnel were blinded to the product. The randomization list was computer‐generated with a block size of 4. The block size was not disclosed to the investigators and allocation was blinded to the participants as well as the site staff. Enrolled participants were instructed to take one capsule per day, before or during a meal, beginning at the day after randomization (d 1).

Throughout the intervention period, patients recorded their daily bowel habits, 3‐day food records, physical activity and compliance. Participants returned for investigational visits after weeks 2 and 4 of the intervention period, which included the completion of PAC‐SYM and PAC‐QoL questionnaires and the collection of study diary information. Fecal sample collection was performed prior to baseline and the end of study visits for microbial profiling analysis.

### 2.3 Preparation of probiotic and placebo capsules

The probiotic product contained four strains: *L. acidophilus* DDS‐1*, B. animalis* subsp. *lactis* UABla‐12*, B. longum* UABl‐14 and *B. bifidum* UABb‐10. Placebo and probiotic capsules (size 1 hypromellose) were prepared in accordance with US Food and Drug Administration good manufacturing practices at UAS Laboratories (Wausau, WI, USA). Probiotic capsules contained a potency of not less than (NLT) 1.5 × 10^10^ colony‐forming units (CFU)/capsule, of which *L. acidophilus, B. animalis* subsp. *lactis, B. longum* and *B. bifidum* were present in CFU/capsule ratios of 44:52:2:2, respectively. They were formulated with lyophilized probiotic blend (57 mg) and rice maltodextrin (280 mg). Placebo capsules were formulated with rice maltodextrin (337 mg). Both capsules contained minimal but identical quantities of fructooligosaccharide (7 mg), magnesium stearate (4 mg) and silica (2 mg). The probiotic and placebo capsules were identical in appearance and taste.

### 2.4 Outcome assessments

The primary outcome was the change in the PAC‐SYM score after week 4 of the intervention, while secondary outcomes included the change in the PAC‐QoL, stool consistency and frequency, microbial profile, strain recovery, and safety parameters over the intervention period. The PAC‐SYM questionnaire^22^ assessed the severity of symptoms related to constipation over the prior 2 weeks. The questionnaire includes 12 items, each rated on a five‐point Likert scale (0 to 4), and is divided into three subcategories. Participants also completed the PAC‐QoL, which assessed quality of life across 28 items and within four domains over the prior 2 weeks. Quality of life scores are registered on a five‐point Likert scale (0 to 4) for each of the 28 items. Higher scores on the PAC‐SYM and PAC‐QoL scales indicate worse symptom severity and quality of life, respectively. The participants self‐administered the international physical activity questionnaire (IPAQ) weekly throughout the study. IPAQ is an instrument designed for the surveillance of physical activity among adults over the prior week. Participants recorded stool frequency, number of complete spontaneous bowel movements (CSBM) and stool consistency (BSS), in a daily bowel habits diary from screening until the end of study. Food records were assessed for their total caloric and macronutrient intake. Compliance was recorded by examining unused study product and confirmed via participants’ diary.

### 2.5 Microbial profiling

Participants collected fecal samples, as close as possible but prior to week 0 and 4 visits, inside a specimen collection hat, which ensured no contact with toilet water or urine. Samples were maintained at −80°C until analysis. Sample DNA isolation, library preparation and microbial profiling of fecal samples were performed by Second Genome (South San Francisco, CA, USA). DNA isolation and DNA quantification utilized the MoBio PowerMag Microbiome kit (Carlsbad, CA, USA) and the Qubit Quant‐iT dsDNA High Sensitivity Kit (Invitrogen, Grand Island, NY, USA), respectively. DNA was subsequently amplified with fusion primers for the 16S V4 region, which incorporated Illumina (San Diego, CA, USA) adapters and indexing barcodes.^23^ Polymerase chain reaction (PCR) products were concentrated, quantified via the Qubit Quant‐iT dsDNA High Sensitivity Kit, pooled equimolar and then sequenced on an Illumina MiSeq (2 ×250 bp paired end).

The paired‐end reads were merged using USEARCH,^24^ and the resulting sequences were compared to an in‐house strain database and Greengenes (version 13.5).^25^ All sequences with an identity ≥99% to a unique strain were assigned a strain operational taxonomic unit (OTU). Sequences were mapped via USEARCH against the OTU representative sequences to calculate strain abundances. The remaining (non‐strain) unique sequences were quality filtered and clustered at 97% by UPARSE, and representative consensus sequences per *de novo* OTU were determined. Each representative OTU sequence was assigned a taxonomic classification using mothur's Bayesian classifier, which was trained against the Greengenes reference database of 16S rRNA gene sequences clustered at 99%.

Alpha diversity (ie, within‐sample diversity) and beta diversity (ie, sample‐to‐sample dissimilarity) metrics were calculated. For the latter, Bray‐Curtis dissimilarity was used to calculate the abundance‐weighted sample pair‐wise differences.^26^ Significant differences among discrete continuous or categorical variables was assessed using permutational analysis of variance.^27^ Metagenomic inference of 16S rRNA sequenced samples was applied as described previously.^28^

### 2.6 Strain recovery and identification

Each quantitative PCR (qPCR) target region was amplified individually from genomic DNA with primer sequences specific to the *L. acidophilus*, *B. animalis subsp. lactis*, *B. longum,* and *B. bifidum* strains (Table S1). The PCR cycling conditions were 1 cycle of 2 minutes at 95°C, 25 cycles of 1 minute at 95°C, 1 minute at 53°C, 15 seconds at 72°C, followed by 10 minutes at 72°C. PCR products for each strain were purified by the QIAquick PCR Purification Kit (Qiagen, Hilden, Germany) and cloned into a plasmid to generate copy number standards that ranged from 20 to 2 000 000 copies for absolute quantification. Plasmid DNA was purified by DNeasy UltraClean Microbial Kit (Qiagen) and quantified by Quant‐iT PicoGreen dsDNA Assay Kit (Invitrogen). The molecular mass for each plasmid was converted into a copy number according to the total length (bp) of the vector and insert. The qPCR reactions were executed using the Brilliant III SYBR Green QPCR Master Mix with Low ROX (Agilent, Santa Clara, CA, USA). The qPCR cycling conditions were 1 cycle of 3 minutes at 95°C, 35 cycles of 20 seconds at 95°C and 1 minute at 62°C. Samples, standards, and a no template control were amplified in triplicate and the mean copy number was used for the comparative analyses. The lowest detected standard was at 200 copies for *L. acidophilus*, *B. animalis* subsp. *lactis*, and *B. bifidum*, and at 20 copies for *B. longum*. Samples below detection were valued at zero.

### 2.7 Statistical analysis

The primary efficacy outcome on which the sample size calculation was based was the change in the PAC‐SYM score between groups from week 0 to week 4 of the intervention period. Assuming alpha equaled to 5% and a power of 80%, the sample size was estimated with a difference in PAC‐SYM of 4.08 units between groups, and a standard deviation (SD) of 6.36 units. The above assumptions were based on prior studies with probiotic^29^ and plant extract^30^ interventions that used the PAC‐SYM score to assess symptoms. Accounting for premature withdrawal, 100 participants were forecasted for randomization, with 50 participants in each group.

The primary and secondary objectives were assessed on the intention‐to‐treat (ITT) population. All the statistical analyses were performed using the R statistical package version 3.2.3 (R Core Team, 2015). Significance tests were two‐sided, and a *P* value of < 0.05 was regarded as significant. Descriptive statistics are presented as mean ± SD, or median and interquartile range (IQR) for continuous variables or as numbers and percentages for qualitative variables. Differences in baseline characteristics were assessed using an independent Student's *t*‐test or a non‐parametric Mann‐Whitney‐Wilcoxon test for continuous variables, or Fisher's exact test for categorical variables, respectively. To test the differences between groups over the intervention period, an analysis of covariance (ANCOVA) was used, taking into account covariates that were identified by multiple linear regression. The dependent variable was the change in the value of the variable (or its logarithmic transform) at the later visit; the intervention was the factor of interest, and the value of the variable (or its logarithmic transform) at the baseline visit was a covariate. The following variables at baseline were prespecified as possible confounders: sex, BMI, age, baseline physical activity, alcohol consumption, and smoking habits. These variables were included in the model as additional covariates, and a reduced set of confounders were identified by stepwise regression. The final reduced model was used for the formal ANCOVA test. For intractably non‐normal variables, the non‐parametric Mann‐Whitney *U* test was used to compare changes from baseline between the placebo and probiotic groups. Microbial profiling and strain recovery assessments utilized a paired Wilcoxon signed‐rank test to identify significant differences between week 0 and week 4 of the intervention period. The Mann‐Whitney *U* test was employed on unpaired genomic delta data.

## 3 RESULTS

### 3.1 Study parameters

**Figure 1:**
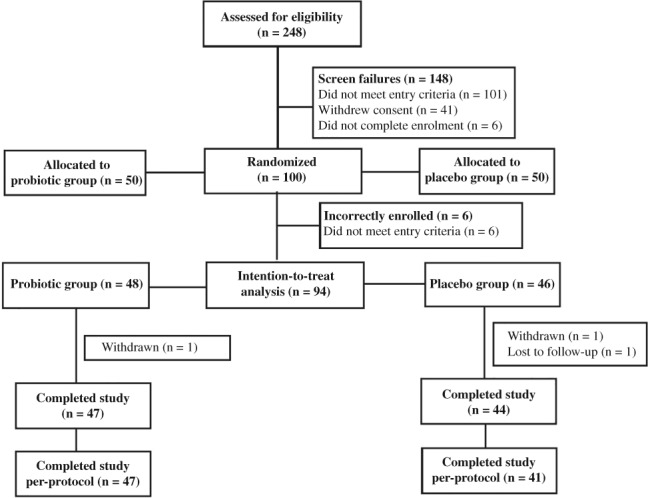
Participants’ enrollment.

A total of 248 participants were screened for the study from May 2015 through April 2016. In all 94 participants were randomized as part of the ITT population, with 46 participants in the placebo group and 48 in the probiotic group (Figure 1). Six participants were incorrectly enrolled with a run‐in average BSS score not less than 3, and were excluded. Two participants withdrew from the study at their request and one was lost to follow‐up. Altogether 88 participants completed the study per‐protocol (PP), with 41 in the placebo group and 47 in the probiotic group. The ITT population reported an overall mean compliance of 98.3% in the placebo group and 99.0% in the probiotic group.

### 3.2 Baseline characteristics of the participants

The baseline characteristics of the ITT population are presented in Table 1, and were shown to be homogeneous in terms of age, sex, body weight, BMI, heart rate, alcohol consumption and smoking status (all *P* > 0.05). Diastolic blood pressure was lower in the probiotic group (9.2 kPa vs 9.7 kPa, *P* = 0.039); however, the absolute difference was similar to that in systolic blood pressure (14.8 kPa vs 15.2 kPa, *P* = 0.255) and was not significant when accounting for multiple comparisons. The ITT population comprised more women (75.5%) than men (24.5%). The participants were deemed to have functional constipation per Rome III criteria. There were no significant differences in parameters of functional constipation, including PAC‐SYM, PAC‐QoL, BSS and CSBM at baseline (all *P* > 0.05).

**Table 1:** Baseline and clinical characteristics of the intention‐to‐treat population

	Placebo (N = 46)	Probiotic (N = 48)	*P* value
Female sex (n, %)	37 (80.4)	34 (70.8)	0.341^a^
Age, y (mean ± SD)	42.9 ± 13.8	44.0 ± 11.3	0.671^b^
Body weight, kg (mean ± SD)	72.5 ± 13.2	71.9 ± 12.5	0.839^b^
BMI, kg/m^2^ (mean ± SD)	26.6 ± 4.5	26.0 ± 3.6	0.456^b^
Systolic BP, kPa (mean ± SD)	15.2 ± 1.6	14.8 ± 1.7	0.255^b^
Diastolic BP, kPa (mean ± SD)	9.7 ± 1.1	9.2 ± 1.1	0.039^b^
Heart rate, bpm (mean ± SD)	70.1 ± 8.7	69.4 ± 9.6	0.736^b^
PAC‐SYM score (mean ± SD)	30.0 ± 7.6	28.7 ± 6.7	0.386^b^
PAC‐QoL score (mean ± SD)	71.7 ± 18.1	71.2 ± 18.3	0.893^b^
BSS run‐in average (mean ± SD)	2.32 ± 0.47	2.19 ± 0.59	0.252^b^
CSBM run‐in average (mean ± SD)	2.68 ± 2.65	2.33 ± 2.19	0.646^c^

Abbreviations: BMI, body mass index; BP, blood pressure; bpm, beats per minute; BSS, Bristol stool scale; CSBM, complete spontaneous bowel movement; PAC‐QoL, patient assessment of constipation ‐ quality of life; PAC‐SYM, patient assessment of constipation ‐ symptoms; SD, standard deviation.

a Between‐group comparison, Fisher's exact test.

b Between‐group comparison, independent Student's *t*‐test.

c Between‐group comparison, Mann‐Whitney *U* test.

### 3.3 Caloric intake and exercise questionnaire

A dietary assessment of macronutrients was performed throughout the study period. The probiotic group showed a higher carbohydrate caloric intake at baseline (*P* = 0.014), but no within‐group changes were observed over the study period (Table 2). Further, no significant differences were observed in intake of total energy, proteins, lipids or water over the intervention period (all *P* > 0.05). Using the IPAQ scoring protocol, weekly physical activity was estimated and categorized as sedentary/light, moderate, or vigorous intensity. Between the two groups, there were no significant differences in the total exercise scores, including vigorous, moderate, and sedentary domains (*P* > 0.05).

**Table 2:** Daily macronutrient intake and physical activity scores

	Placebo (N = 46)	Probiotic (N = 48)	*P* value
At week	Mean ± SD	Mean ± SD	
Energy (kJ)			
0	7225.8 ± 2000.0	7665.1 ± 2192.4	0.282^a^
2	7112.8 ± 2217.5	7204.8 ± 2129.7	0.761^a^
4	6932.9 ± 2184.1	7518.6 ± 2000.0	0.124^a^
Protein (kJ)			
0	1376.5 ± 531.4	1238.5 ± 485.3	0.227^a^
2	1380.7 ± 548.1	1175.7 ± 397.5	0.104^a^
4	1276.1 ± 422.6	1255.2 ± 389.1	1.000^a^
Carbohydrates (kJ)			
0	3334.7 ± 1029.3	3907.9 ± 1271.9	0.014^a^
2	3276.1 ± 1159.0	3615.0 ± 1255.2	0.126^a^
4	3276.1 ± 1251.0	3727.9 ± 1251.0	0.074^a^
Lipids (kJ)			
0	2410.0 ± 928.8	2410.0 ± 853.5	0.913^a^
2	2326.3 ± 1000.0	2301.2 ± 861.9	0.951^a^
4	2288.7 ± 949.8	2418.4 ± 744.8	0.204^a^
Water (L)			
0	1.23 ± 0.59	1.30 ± 0.57	0.518^a^
2	1.25 ± 0.62	1.35 ± 0.59	0.436^a^
4	1.23 ± 0.59	1.35 ± 0.62	0.304^a^
Total MET score			
0	3969 ± 3669	4781 ± 4719	0.250^b^
2	4270 ± 5012	4227 ± 5363	0.593^b^
4	3565 ± 4284	3889 ± 3271	0.208^b^

Abbreviations: kJ, kilojoules; MET, metabolic equivalent; SD, standard deviation.

a Between‐group comparison, independent Student's *t*‐test.

b Between‐group comparison, Mann‐Whitney *U* test.

### 3.4 Questionnaire scores

There was no significant difference in the PAC‐SYM total score at week 4 (*P* > 0.05) when comparing probiotic group with the placebo group (Table 3). Participants supplemented with the probiotic showed significant within‐group reductions of 5.8 units, or 20.4%, after 4‐weeks of intervention (*P* < 0.001). However, significant within‐group reductions (*P* < 0.001) were observed in the placebo group as well. Similarly, PAC‐SYM abdominal, rectal, and stool subscales (Table S2) showed significant within‐group reductions over the intervention period of 1.9 units (18.0%), 1.1 units (15.0%) and 2.8 units (21.7%), respectively (*P* < 0.001), in probiotic‐treated participants. However, similar within‐group reductions (*P* < 0.001) were observed in the placebo group. The assessment of the PAC‐QoL total score did not show significant differences between groups at week 4 (*P* > 0.05), with both groups showing significant within‐group reductions (*P* < 0.001). Similarly PAC‐QoL physical discomfort, psychological discomfort, worries and concerns, and satisfaction subscales (Table S2) were significantly reduced at week 4 in both the probiotic and placebo groups (*P* < 0.01).

**Table 3:** Changes from baseline in key parameters of functional constipation in participants receiving placebo or probiotic capsules

	Placebo (N = 46)		Probiotic (N = 48)		*P* value (between groups)
Score per week	Mean ± SD	*P* value (within group)	Mean ± SD	*P* value (within group)	
PAC‐SYM					
Week 0	30.0 ± 7.6		28.7 ± 6.7		
AbsΔ (2)	−4.6 ± 7.2	<0.001^a^	−3.5 ± 6.0	<0.001^a^	0.634^b^
AbsΔ (4)	−7.0 ± 8.1	<0.001^a^	−5.8 ± 6.3	<0.001^a^	0.866^b^
PAC‐QoL score					
Week 0	71.7 ± 18.1		71.2 ± 18.3		
AbsΔ (2)	−9.7 ± 16.0	<0.001^a^	−9.2 ± 12.1	<0.001^a^	0.974^b^
AbsΔ (4)	−15.8 ± 20.5	<0.001^a^	−13.1 ± 14.2	<0.001^a^	0.701^b^
BSS average					
Week 0	2.32 ± 0.47		2.19 ± 0.59		
AbsΔ (1)	0.21 ± 0.76	0.072^a^	0.66 ± 0.89	<0.001^a^	0.030^b^
AbsΔ (2)	0.68 ± 0.97	<0.001^a^	0.71 ± 0.76	<0.001^a^	0.984^b^
AbsΔ (3)	0.76 ± 1.11	<0.001^a^	0.77 ± 0.81	<0.001^a^	0.761^b^
AbsΔ (4)	0.79 ± 1.02	<0.001^a^	0.78 ± 0.89	<0.001^a^	0.856^b^
CSBM average					
Week 0	2.68 ± 2.65		2.33 ± 2.19		
AbsΔ (1)	0.21 ± 2.60	0.575^c^	1.29 ± 2.38	<0.001^c^	0.057^d^
AbsΔ (2)	1.03 ± 2.61	0.009^c^	1.86 ± 2.80	<0.001^c^	0.310^d^
AbsΔ (2)	1.60 ± 2.58	<0.001^c^	2.21 ± 3.09	<0.001^c^	0.583^d^
AbsΔ (4)	0.90 ± 2.90	0.023^c^	2.00 ± 3.10	<0.001^c^	0.166^d^

Abbreviations: AbsΔ, absolute change; BSS, Bristol stool scale; CSBM, complete spontaneous bowel movement; PAC‐QoL, patient assessment of constipation ‐ quality of life; PAC‐SYM, patient assessment of constipation ‐ symptoms; SD, standard deviation.

a Within‐group comparison, paired Student's *t*‐test.

b Between‐group comparison, ANCOVA.

c Within‐group comparison, signed‐rank test.

d Between‐group comparison, Mann‐Whitney *U*‐test.

### 3.5 Bowel habits

Participants receiving the probiotic showed a significant improvement in average stool consistency compared to the placebo group (*P* = 0.03), after the first week of intervention (Table 3). The mean BSS score of the probiotic group increased by 0.66 units, or 34%, approaching a normal stool consistency after 1 week of supplementation. In contrast, the BSS score of the placebo group increased by 0.21 units, or 14%, after 1 week of supplementation. However, no between‐group effects were observed after weeks 2, 3, and 4 (*P* > 0.05) as both groups approached a normal stool consistency at the end of study. While a placebo response in stool consistency was observed, this response was delayed compared with participants receiving the probiotic product.

Between the two groups, there were no significant differences in average stool frequency, as assessed by CSBM per week. However, there was a trend for participants receiving the probiotic to have a greater number of CSBM, as compared with the placebo group, over the first week of intervention (*P* = 0.057). As compared to baseline, the mean weekly CSBM of the probiotic group increased by 1.29 units (*P* < 0.001), 1.86 units (*P* < 0.001), 2.21 units (*P* < 0.001) and 2.00 units (*P* < 0.001) after weeks 1, 2, 3, and 4, respectively. There was a nearly 2‐fold increase in weekly CSBM over the course of the study in participants receiving the probiotic. In comparison, the mean weekly CSBM of the placebo group increased by 0.21 units (*P* = 0.575), 1.03 units (*P* = 0.009), 1.60 units (*P* < 0.001) and 0.90 units (*P* = 0.023), after weeks 1, 2, 3, and 4, respectively. A subgroup analysis was also performed in participants with severe or very severe hard stool symptoms at baseline (Table S3). The stool consistency (BSS) and frequency (CSBM) of the probiotic group was significantly improved, compared with the placebo group after the first week of intervention (*P* < 0.05). However, no between‐group differences were observed at later time points.

### 3.6 Microbial profiling

**Figure 2:**
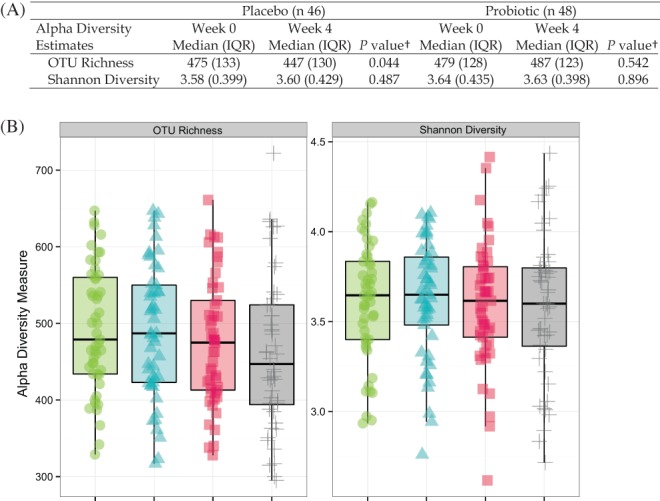
Alpha diversity estimates, represented by operational taxonomic unit richness or Shannon diversity. (A) Median and interquartile range (IQR) in participants receiving placebo or probiotic capsules. †Wilcoxon signed‐rank test. (B) Box plot representing distribution of participants receiving placebo or probiotic capsules. 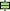 probiotic, week 0; 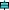 probiotic, week 4; 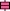 placebo, week 0; 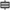 placebo, week 4

Probiotic group samples had similar alpha diversity measures (OTU richness and Shannon diversity) at week 0 and week 4. In contrast, the placebo group samples had significantly lower OTU richness at week 4 as compared with baseline while Shannon diversity, which considered richness and evenness of OTU, did not change (Figure 2).

**Figure 3:**
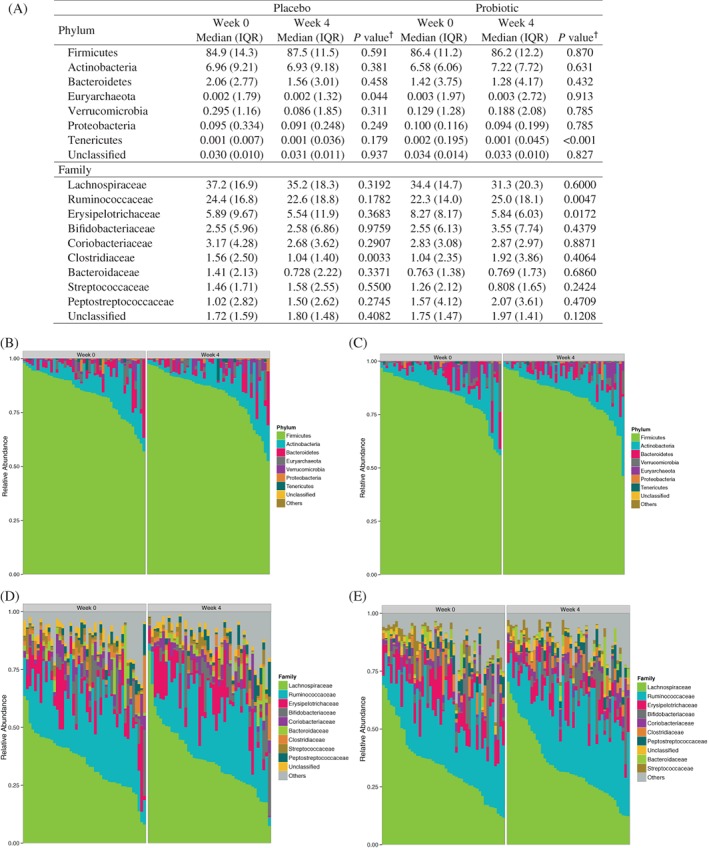
Relative abundance profiles of the most abundant phyla and families. (A) Median and interquartile range (IQR) in participants receiving placebo or probiotic capsules. †Wilcoxon signed‐rank test. Proportional phylum and family abundance in participants receiving (B,D) placebo or (C,E) probiotic capsules

Figure 3 shows the most abundant phyla and families in the participants treated with placebo and probiotic. The placebo group samples had a higher relative abundance of Euryarchaeota (*P* = 0.044) at week 4 as compared with week 0, while the probiotic group samples had a lower relative abundance of Tenericutes (*P* < 0.001) at week 4 than week 0. The abundance of the three most abundant taxa at the phylum level, Firmicutes, Actinobacteria and Bacteroidetes, did not differ significantly over time in both groups. Among the most abundant families, probiotic group samples had significantly higher relative abundance of Ruminococcaceae (*P* = 0.0047) and lower relative abundance of Erysipelotrichaceae (*P* = 0.0172) at week 4 compared to week 0. Within the probiotic group, two Ruminococcaceae genera, including *Ruminococcus*, were significantly more abundant in the week 4 samples. Placebo group samples showed similar abundance profiles at baseline and end‐point, with the exception of Clostridiaceae, which decreased over the course of the study (*P* = 0.0033). The increased abundance of Ruminococcaceae and decreased abundance of Tenericutes in the probiotic group and the decreased abundance of Clostridiaceae in the placebo group met the significance threshold after adjusting for multiple comparison testing.

Regarding beta diversity, samples did not cluster or separate according to time point in either group. However, it was determined that sex (*P* = 0.012) and BSS score (*P* = 0.001), contributed to overall beta diversity. Dietary data from the food records was assessed and was found to not be correlated to the beta diversity of the samples.

### 3.7 Metagenomic inference

There were no significant differences in Kyoto Encyclopedia of Genes and Genomes (KEGG) pathway abundances between week 0 and week 4 in the probiotic or placebo groups. Among KEGG orthologs, in participants receiving probiotic, 10 features had an unadjusted *P* value of < 0.05 and an absolute fold‐change of greater than 2, with nine of the 10 features showing an enrichment at week 4 compared with week 0 (Table S4). However, no features passed multiple testing correction. In participants receiving placebo no features had an absolute fold‐change of greater than 2.

### 3.8 Strain recovery

**Figure 4:**
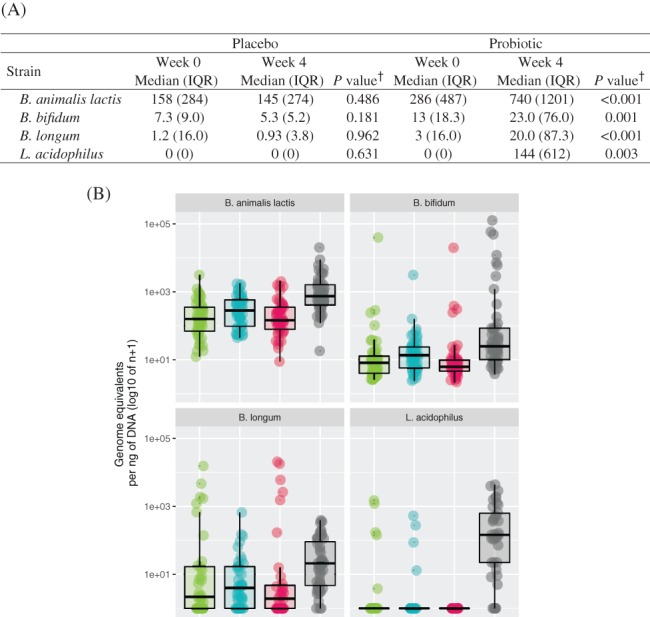
Total genome equivalents of each strain, per ng of DNA, by intervention and visit. (A) Median and interquartile range (IQR) in participants receiving placebo or probiotic capsules. †Wilcoxon signed‐rank test. (B) Box plot representing the total genome equivalents observed in participants receiving placebo or probiotic capsules. 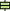 placebo, week 0; 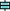 probiotic, week 0; 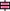 placebo, week 4; 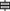 probiotic, week 4

Probiotic group samples had a significant increase in the number of genome equivalents for all four strains evaluated. Among probiotic group samples, *B. animalis* subsp. *lactis* (*P* < 0.001), *B. bifidum* (*P* = 0.001), *B. longum* (*P* < 0.001) and *L. acidophilus* (*P* = 0.003) were significantly more abundant in week 4 as compared with week 0 (Figure 4). Participants who received the placebo had no detectable shift in the strains evaluated.

### 3.9 Safety profile

There were no significant differences in hematology (Table S5) or biochemical safety parameters (Table S6) between placebo or probiotic groups over the study period. Mean values were within clinical reference ranges pre and post‐intervention. Additionally, blood pressure, heart rate, weight and BMI remained similar throughout the study (Table S7). There were a total of 25 adverse events, with 14 reported in the placebo group and 11 reported in the probiotic group (Table S8). A total of nine adverse events, primarily gastrointestinal, were found to be possibly related to the study product, with five reported in the placebo group and four reported in the probiotic group.

## 4 DISCUSSION

The present study was a randomized, double‐blind, placebo‐controlled trial to assess a probiotic product consisting of *L. acidophilus* DDS‐1*, B. animalis* subsp. *lactis* UABla‐12*, B. longum* UABl‐14, and *B. bifidum* UABb‐10, in participants with functional constipation. Previously, this probiotic blend was shown to improve irritable bowel syndrome outcomes in an open‐label study.^21^ Further, *L. acidophilus* DDS‐1, alone or in combination with *B. animalis* subsp. *lactis* UABla‐12, was shown to support abdominal symptom relief for lactose intolerance,^31^ reduce the severity of atopic dermatitis^32^ and reduce the severity and duration of acute viral respiratory tract infection^33^ in randomized controlled trials.

The current study enrolled on average middle‐aged, predominately female participants, with normal to overweight BMI, representing a typical cross‐section of the functional constipation population.^34^ Participants in the probiotic group demonstrated a faster normalization of stool frequency and consistency, in comparison with placebo, with most participants achieving a normalized profile within 1 week of intervention. Further, the probiotic was shown to be well tolerated according to biochemical, hematological, and adverse events profiles. However, results did not show a significant difference in PAC‐SYM or PAC‐QoL questionnaire scores, with both treatments showing an approximate 20% improvement in both assessments. Improvements in bowel profile in the absence of a corresponding effect in PAC‐SYM questionnaire scores have previously been reported.^29^ Further, symptomology was assessed only at time points in which there were no significant between‐group differences in bowel habits. The mean BSS score of the probiotic group increased by 0.66 units, or 0.45 units compared with the placebo within the first week of supplementation. This was in accordance with a meta‐analysis of studies that administered *B. animalis* subsp. *lactis*, which showed significant improvement in stool consistency (standardized mean difference [SMD]: +0.46), albeit with high heterogeneity.^15^ In contrast, a similar assessment of studies that administered the *L. casei* Shirota strain failed to show a significant amelioration in stool consistency (SMD: +0.26).^15^ The CSBM, defined as a spontaneous bowel movement associated with a sensation of complete evacuation, showed a near doubling in the probiotic group over the study period. However, this effect was not significantly different from the placebo. Normal stool frequency has been reported to range from three to 21 total bowel movements per week,^35^ of which a smaller number would be CSBMs. In the current study, an increase in CSBM from 2.3 to 4.3 per week suggests a normalization of bowel frequency in the probiotic group. In contrast, in a systematic review, laxatives and fiber were shown to increase stool frequency by an average of 1.4 total bowel movements per week.^36^ A recent study with chicory inulin, which has an approved European Food Safety Authority health claim for maintainence of normal defecation, similarly showed an improved stool frequency of 4.0 vs 3.0 bowel movements per week in constipated subjects.^37^ This was also in line with a meta‐analysis of probiotic studies that demonstrated increased stool frequency, by an average of 1.3 bowel movements per week, compared with a placebo.^15^ Interestingly, the reported effect in the pooled analysis coincided with a reduction in whole gut transit time by 12.4 h,^15^ suggesting that the probiotic group in the current study may have experienced improved intestinal transit. However, this remains to be determined.

The gastrointestinal microbiota has been reported to play a role in gut motility. Early studies in germ‐free rats demonstrated delayed gastric emptying and colonic transit in comparison with their conventional counterparts.^9^ Further, colonizing germ‐free rats with *L. acidophilus* and *B. bifidum* helped normalize intestinal transit.^38^ The mechanisms for this are likely to be varied and may involve end‐products of bacterial fermentation as well as modulation of the neuroendocrine function or the immune response.^39^ Individuals with functional constipation have been shown to have increased pathogenic bacteria or fungi at the expense of *Bifidobacterium* and *Lactobacillus*.^40^ Several Firmicute genera, including *Faecalibacterium*, *Roseburia,* and *Coprococcus*, have been reported to be directly correlated with colonic transit.^7^ *Faecalibacterium*, in particular, is among the most plentiful butyrate producers.^41^ In contrast, Bacteroidetes, notably *Bacteroides,* were shown to be inversely correlated with colonic transit,^7^ as well as dietary fiber intake.^42^

The current study demonstrated a significantly higher relative abundance of Ruminococcaceae within the probiotic group over the study period. In contrast, the placebo group showed a significant decrease in Clostridiaceae. Ruminococcaceae, the second most abundant Firmicutes family in gut environment,^43^ persist in communities of fibrolytic organisms and are well adapted to utilize or degrade complex and otherwise indigestible plant material.^44^ As a result, they help generate short‐chain fatty acids (SCFA) such as butyrate, acetate, and propionate, which can be used for energy by the host.^45^ The Ruminococcaceae family is commonly associated with Clostridium clusters IV/XIVa, essential bacteria that produce SCFA.^46^ Further, Ruminococcaceae abundance has been shown to be positively correlated with BSS scores and faster intestinal transit.^47^ SCFA have been implicated in gut motility, among other potential mechanisms, by increasing the intestinal release of 5‐hydroxytryptamine^48^ or stimulating ileal and colonic smooth muscle contractility.^9^ Several Firmicute genera shown previously to correlate with faster colonic transit, are similarly butyrate producers.^7^

In the probiotic group the increase in Ruminococcaceae appeared to coincide with a decrease in Erysipelotrichaceae. This is potentially of interest, as members of the latter appear to be enriched in metabolic disorders^49^ and inflammation‐related gastrointestinal disorders^50^ in comparison with healthy controls. In the context of the current study, the increase in Ruminococcaceae and corresponding decrease in Erysipelotrichaceae is worth further investigation, particularly as it may relate to improved gut motility or decreased inflammation. Tenericutes was also lower in week 4 probiotic samples as compared to week 0; however, this shift is likely to be of less clinical significance due to its very low abundance levels. The current literature does not support a universal abundance cut‐off for biological significance and therefore lower abundant phyla and families were reported here. For instance, biological significance may depend on the complexity and type of microbial community, and a rare member may carry out a key function in the community. It should also be noted that while dietary effects on the intestinal microbiota have previously been demonstrated,^51^ the current trial found no contribution of the dietary data to the beta diversity of the samples.

While probiotic products are ultimately supported by randomized controlled trials demonstrating health benefits, it is also of importance to assess the presence of the live organisms post‐transit.^20^ In the current study, participants receiving the probiotic had a significant increase in number of genome equivalents for all four strains evaluated, while those who received the placebo had no detectable shift in the strains evaluated. It should be noted that the probiotic product did not significantly disrupt the microbiota composition, in line with a recent systematic review of probiotics which reported no effects on alpha diversity, richness, or evenness.^52^ In the current study, richness was maintained in the probiotic group, while simultaneously decreasing in the placebo group. However, no changes were observed when considering richness and evenness. No significant changes in beta diversity were found either, also in line with the systematic review,^52^ although beta diversity did appear to be positively correlated with stool consistency.

Placebo response can be high in participants with bowel disorders, potentially leading to altered outcomes reported by the patient, and the power calculation for the current study may have underestimated this response. The study did not include a placebo run‐in period. The inclusion of such a period may have helped control the placebo response of participants entering the trial by excluding high responders.^53^, ^54^ Longer run‐in phases have also been associated with a less pronounced placebo response.^55^ Second, the PAC‐SYM score was not included as part of the primary inclusion criteria. Aligning the study inclusion with the study outcomes has been recommended, due to significant variation in functional constipation outcomes.^56^ These limitations in the current study design may have ultimately affected its ability to differentiate between groups. As it relates to microbial profiling, the study did not assess colonic mucosal microbiota, which may present differences from fecal microbiota in functional constipation.^7^ Also, while the study employed Rome III criteria, Rome IV criteria have since been released, which takes the view that functional bowel disorders exist on a continuum instead of as discrete disorders.^57^

In summary, the multi‐strain probiotic product was shown to be well tolerated but did not significantly affect symptomology, in part due to a significant placebo response. Nevertheless, the probiotic exhibited potential in modulating bowel habits and microbial profile in participants with functional constipation.

## CONFLICT OF INTEREST

This study was sponsored by UAS Laboratories. Christopher J. Martoni and Gregory Leyer are employees of UAS Laboratories but were not involved in the study conduct, data management or statistical analysis.

## Supporting information


**Supplemental Table 1.** Quantitative polymerase chain reaction (qPCR) primer sequences to assess fecal recovery and identification of probiotic strains
**Supplemental Table 2.** Patient assessment ofconstipation ‐ symptoms (PAC‐SYM) and patient assessment of constipation ‐ qualityof life (PAC‐QoL) subscale scores over the intervention period in participants receiving placebo or probiotic capsules.
**Supplemental Table 3.** Changes from baseline in stool consistency and frequency in subgroup of participants with severe or very severe hard stool symptoms at baseline.
**Supplemental Table 4.** KEGG pathway abundances with an absolute fold‐change greater than two over the intervention period.
**Supplemental Table 5.** Hematology data at screening and after intervention period.
**Supplemental Table 6.** Biochemistry data at screening and after intervention period.
**Supplemental Table 7.** Blood pressure (BP), heart rate, and weight data at screening and after intervention period.
**Supplemental Table 8.** Summary of adverse events (AEs) over entire study period (n).
