# Supplementary material for: Impact of a probiotic product on bowel habits and microbial profile in participants with functional constipation: A randomized controlled trial
Source: J Dig Dis. 2019 Aug 1;20(9):435–46. doi: 10.1111/1751-2980.12797 (PMC6851827; doi:10.1111/1751-2980.12797)
Supplement: Supplementary file 1 — Supplemental Table 1. Quantitative polymerase chain reaction (qPCR) primer sequences to assess fecal recovery and identification of probiotic strains Supplemental Table 2. Patient assessment ofconstipation ‐ symptoms (PAC‐SYM) and patient assessment of constipation ‐ qualityof life (PAC‐QoL) subscale scores over the intervention period in participants receiving placebo or probiotic capsules. Supplemental Table 3. Changes from baseline in stool consistency and frequency in subgroup of participants with severe or very severe hard stool symptoms at baseline. Supplemental Table 4. KEGG pathway abundances with an absolute fold‐change greater than two over the intervention period. Supplemental Table 5. Hematology data at screening and after intervention period. Supplemental Table 6. Biochemistry data at screening and after intervention period. Supplemental Table 7. Blood pressure (BP), heart rate, and weight data at screening and after intervention period. Supplemental Table 8. Summary of adverse events (AEs) over entire study period (n). [file CDD-20-435-s001.docx]

**Supplemental Table 1.** Quantitative polymerase chain reaction (qPCR) primer sequences to assess fecal recovery and identification of probiotic strains

|  | qPCR primer sequences (5’→3’) |
| --- | --- |
| *Lactobacillus acidophilus* | F: CCGGTTAATAAAATCTTTTCACCTTG  R: GCAGTTATTAATCGTGATTTGCATATAAATT |
| *Bifidobacterium animalis* subsp. *lactis* | F: CTTCCCAGAAGGCCGGGT  R: CGAGGCCACGGTGCTCATATAGA |
| *Bifidobacterium longum* | F: ATCACCGCAATCGTTGGAATAGC  R: TTGCAACCACCACTAGAATCTGC |
| *Bifidobacterium bifidum* | F: GGGTGGTAAGGGAAGGGTTAGGT  R: GACGAAGCAAGACCGAAGCAAG |

F, forward; R, reverse.

**Supplemental Table 2.** Patient assessment of constipation - symptoms (PAC-SYM) and patient assessment of constipation - quality of life (PAC-QoL) subscale scores over the intervention period in subjects receiving placebo or probiotic capsules.

|  | Placebo (N = 46) | |  | Probiotic (N = 48) | |  | *P* value (between groups) |
| --- | --- | --- | --- | --- | --- | --- | --- |
|  | Mean ± SD | *P* value (within group) |  | Mean ± SD | *P* value (within group) |  |  |
| **PAC-SYM subscales** |  |  |  |  |  |  |  |
| Abdominal |  |  |  |  |  |  |  |
| Week 0 | 9.15 ± 2.72 | -- |  | 9.02 ± 2.86 | -- |  |  |
| Week 2 | 8.20 ± 2.90 | 0.025^§^ |  | 8.40 ± 3.50 | 0.075^§^ |  | 0.784^¶^ |
| Week 4 | 7.65 ± 3.43 | <0.001^§^ |  | 7.15 ± 2.37 | <0.001^§^ |  | 0.909^¶^ |
| Rectal |  |  |  |  |  |  |  |
| Week 0 | 5.54 ± 2.24 | -- |  | 5.31 ± 2.28 | -- |  |  |
| Week 2 | 4.63 ± 1.81 | 0.001^§^ |  | 4.71 ± 1.88 | 0.047^§^ |  | 0.928^¶^ |
| Week 4 | 4.48 ± 1.68 | <0.001^§^ |  | 4.21 ± 1.84 | <0.001^§^ |  | 0.176^¶^ |
| Stool |  |  |  |  |  |  |  |
| Week 0 | 15.3 ± 3.9 | -- |  | 14.3 ± 3.6 | -- |  |  |
| Week 2 | 12.5 ± 3.9 | <0.001^†^ |  | 12.1 ± 4.5 | <0.001^†^ |  | 0.708^‡^ |
| Week 4 | 10.8 ± 4.5 | <0.001^†^ |  | 11.5 ± 5.1 | <0.001^†^ |  | 0.126^‡^ |
| **PAC-QoL subscales** |  |  |  |  |  |  |  |
| Physical discomfort |  |  |  |  |  |  |  |
| Week 0 | 10.5 ± 3.0 | -- |  | 10.3 ± 2.9 | -- |  |  |
| Week 2 | 8.7 ± 3.1 | <0.001^†*^ |  | 9.0 ± 3.2 | 0.003^†*^ |  | 0.624^‡*^ |
| Week 4 | 7.7 ± 3.3 | <0.001^†*^ |  | 8.2 ± 3.5 | <0.001^†*^ |  | 0.556^‡*^ |
| Psychological |  |  |  |  |  |  |  |
| Week 0 | 15.8 ± 6.3 | -- |  | 15.7 ± 6.4 | -- |  |  |
| Week 2 | 13.3 ± 5.6 | <0.001^†^ |  | 12.9 ± 5.4 | <0.001^†^ |  | 0.780^¶^ |
| Week 4 | 11.7 ± 5.1 | <0.001^†^ |  | 12.1 ± 5.1 | <0.001^†^ |  | 0.652^¶^ |
| Worries and concerns |  |  |  |  |  |  |  |
| Week 0 | 26.6 ± 8.5 | -- |  | 26.2 ± 8.9 | -- |  |  |
| Week 2 | 22.4 ± 8.6 | <0.001^†*^ |  | 23.1 ± 8.8 | <0.001^†*^ |  | 0.335^‡*^ |
| Week 4 | 20.7 ± 8.0 | <0.001^†*^ |  | 21.0 ± 8.5 | <0.001^†*^ |  | 0.933^‡*^ |
| Satisfaction |  |  |  |  |  |  |  |
| Week 0 | 18.7 ± 3.9 | -- |  | 19.0 ± 3.9 | -- |  |  |
| Week 2 | 17.6 ± 4.1 | 0.174^†^ |  | 17.0 ± 4.9 | 0.002^†^ |  | 0.361^‡^ |
| Week 4 | 15.7 ± 5.4 | 0.005^†^ |  | 16.8 ± 5.4 | 0.009^†^ |  | 0.504^‡^ |

^†^Within-group comparison, paired Student’s *t*-test.

^‡^Between-group comparison, ANCOVA.

^§^Within-group comparison, Signed-rank test.

^¶^Between-group comparison, Mann-Whitney U-test.

*Logarithmic transformation prior to analysis.

**Supplemental Table 3.** Changes from baseline in stool consistency and frequency in subgroup of subjects with severe or very severe hard stool symptoms at baseline.

|  | Placebo (N = 15) | |  | Probiotic (N = 11) | |  | *P* value (between groups) |
| --- | --- | --- | --- | --- | --- | --- | --- |
|  | Mean ± SD | *P* value (within group) |  | Mean ± SD | *P* value (within group) |  |  |
| BSS average |  |  |  |  |  |  |  |
| Week 0 | 2.28 ± 0.53 | -- |  | 1.74 ± 0.51 | -- |  |  |
| AbsΔ (Week 1) | 0.00 ± 0.76 | 0.991^†^ |  | 1.03 ± 1.45 | 0.041^†^ |  | 0.049^‡^ |
| AbsΔ (Week 2) | 0.33 ± 1.09 | 0.259^†^ |  | 0.67 ± 0.95 | 0.041^†^ |  | 0.364^‡^ |
| AbsΔ (Week 3) | 0.61 ± 1.07 | 0.043^†^ |  | 0.36 ± 0.81 | 0.172^†^ |  | 0.604^‡^ |
| AbsΔ (Week 4) | 0.76 ± 1.14 | 0.022^†^ |  | 0.44 ± 0.52 | 0.020^†^ |  | 0.286^‡^ |
| CSBM average |  |  |  |  |  |  |  |
| Week 0 | 1.97 ± 2.19 | -- |  | 1.05 ± 1.39 | -- |  |  |
| AbsΔ (Week 1) | -0.43 ± 2.01 | 0.134^§^ |  | 1.68 ± 2.15 | 0.043^§^ |  | 0.016^¶^ |
| AbsΔ (Week 2) | 0.30 ± 1.71 | 0.525^§^ |  | 2.05 ± 3.15 | 0.106^§^ |  | 0.388^¶^ |
| AbsΔ (Week 2) | 1.57 ± 2.21 | 0.027^§^ |  | 2.05 ± 2.78 | 0.058^§^ |  | 0.835^¶^ |
| AbsΔ (Week 4) | 0.80 ± 2.50 | 0.299^§^ |  | 2.30 ± 3.50 | 0.057^§^ |  | 0.349^¶^ |

Abbreviations: AbsΔ, absolute change; BSS, bristol stool scale; CSBM, complete spontaneous bowel movement; SD, standard deviation.

^†^Within-group comparison, paired Student’s *t*-test.

^‡^Between-group comparison, ANCOVA.

^§^Within-group comparison, signed-rank test.

^¶^Between-group comparison, Mann-Whitney U-test.

**Supplemental Table 4.** KEGG pathway abundances with an absolute fold change greater than 2 over the intervention period

| KO/description | Fold change | *P* value^†^ | *P* value (adjusted) | Group | Enriched  in |
| --- | --- | --- | --- | --- | --- |
| K00897; aphA; kanamycin kinase | 2.609 | 0.049 | >0.05 | Probiotic | Week 4 |
| K0157; oxc; oxalyl-CoA decarboxylase | 2.993 | 0.006 | >0.05 | Probiotic | Week 4 |
| K07693; desR; two-component system, NarL family, response regulator DesR | -2.18 | 0.002 | >0.05 | Probiotic | Week 0 |
| K07749; frc; formyl-CoA transferase | 2.834 | 0.005 | >0.05 | Probiotic | Week 4 |
| K08256; pimA; phosphatidylinositol alphamannosyltransferase | 5.639 | <0.001 | >0.05 | Probiotic | Week 4 |
| K10242; cebG; cellobiose transport system permease protein | 5.587 | <0.001 | >0.05 | Probiotic | Week 4 |
| K14153; thiDE; hydroxymethyl-pyrimidine kinase/phospho-methylpyrimidine kinase/ thiamine-phosphate diphos- phorylase | 5.626 | <0.001 | >0.05 | Probiotic | Week 4 |
| K19131; csb1; CRISPR-associated protein Csb1 | 3.327 | <0.001 | >0.05 | Probiotic | Week 4 |
| K19132; csb2; CRISPR-associated protein Csb2 | 3.327 | <0.001 | >0.05 | Probiotic | Week 4 |
| K19133; csb3; CRISPR-associated protein Csb3 | 3.326 | <0.001 | >0.05 | Probiotic | Week 4 |

^†^Wilcoxon signed rank test.

**Supplemental Table 5.** Hematology data at screening and after intervention period

| Mean ± SD | Placebo (N = 50) | Probiotic (N = 50) | *P* value (between groups)^†^ |
| --- | --- | --- | --- |
| Hemoglobin (g/L) |  |  |  |
| Screening | 136.3 ± 11.0 | 136.2 ± 10.8 | 0.985 |
| End of study | 134.1 ± 11.9 | 133.7 ± 10.5 | 0.845 |
| Hematocrit |  |  |  |
| Screening | 0.400 ± 0.028 | 0.401 ± 0.026 | 0.864* |
| End of study | 0.397 ± 0.028 | 0.394 ± 0.026 | 0.676* |
| White blood cell (×10^9^/L) |  |  |  |
| Screening | 5.90 ± 1.38 | 5.66 ± 1.16 | 0.340 |
| End of study | 5.74 ± 1.46 | 5.64 ± 1.50 | 0.715 |
| Red blood cell (×10^12^/L) |  |  |  |
| Screening | 4.53 ± 0.35 | 4.54 ± 0.36 | 0.973 |
| End of study | 4.50 ± 0.36 | 4.47 ± 0.35 | 0.742 |
| Platelet (×10^9^/L) |  |  |  |
| Screening | 261 ± 51 | 252 ± 56 | 0.416 |
| End of study | 266 ± 55 | 256 ± 56 | 0.371 |

Abbreviation: SD, standard deviation.

^†^Between-group comparison, independent Student’s *t*-test

*Logarithmic transformation prior to analysis

**Supplemental Table 6.** Biochemistry data at screening and after intervention period.

| Mean ± SD | Placebo (N = 50) | Probiotic (N = 50) | *P* value (between groups)^†^ |
| --- | --- | --- | --- |
| Creatinine (μmol/L) |  |  |  |
| Screening | 66.7 ± 10.5 | 67.8 ± 10.8 | 0.617^†^* |
| End of study | 66.0 ± 9.9 | 67.3 ± 10.3 | 0.525^†^* |
| Sodium (mmol/L) |  |  |  |
| Screening | 141.9 ± 2.1 | 141.3 ± 2.2 | 0.173^†^ |
| End of study | 141.6 ± 2.0 | 140.9 ± 1.9 | 0.082^†^ |
| Potassium (mmol/L) |  |  |  |
| Screening | 4.60 ± 0.41 | 4.54 ± 0.44 | 0.527^†^ |
| End of study | 4.57 ± 0.44 | 4.42 ± 0.40 | 0.071^†^ |
| Chloride (mmol/L) |  |  |  |
| Screening | 106.0 ± 1.7 | 106.3 ± 2.6 | 0.527^†^ |
| End of study | 106.2 ± 2.4 | 105.6 ± 2.8 | 0.284^†^ |
| Bilirubin (μmol/L) |  |  |  |
| Screening | 11.3 ± 7.2 | 11.6 ± 6.4 | 0.605^†^* |
| End of study | 9.7 ± 5.2 | 11.0 ± 6.0 | 0.134^†^* |
| Aspartate Transaminase (U/L) |  |  |  |
| Screening | 22.7 ± 5.8 | 25.0 ± 7.0 | 0.088^‡^ |
| End of study | 22.9 ± 6.2 | 26.7 ± 12.4 | 0.096^‡^ |
| Alanine Transaminase (U/L) |  |  |  |
| Screening | 22.4 ± 10.5 | 24.3 ± 10.9 | 0.332^‡^ |
| End of study | 22.5 ± 9.4 | 25.3 ± 13.7 | 0.433^‡^ |
| Gamma-Glutamyltransferase (U/L) |  |  |  |
| Screening | 18.8 ± 14.1 | 17.0 ± 13.0 | 0.421^‡^ |
| End of study | 18.3 ± 12.2 | 16.3 ± 10.5 | 0.338^‡^ |

Abbreviation: SD, standard deviation.

^†^Between-group comparison, independent Student’s *t*-test.

^‡^Between-group comparison, Mann-Whitney U-test.

*Logarithmic transformation prior to analysis.

**Supplemental Table 7.** Blood pressure (BP), heart rate and weight data at screening and after intervention period

| Mean ± SD | Placebo (N = 50) | Probiotic (N = 50) | *P* value (between groups)^†^ |
| --- | --- | --- | --- |
| Systolic BP (kPa) |  |  |  |
| Screening | 15.3 ± 1.9 | 14.8 ± 1.7 | 0.176 |
| End of study | 15.1 ± 1.5 | 14.9 ± 1.6 | 0.393 |
| Diastolic BP (kPa) |  |  |  |
| Screening | 9.6 ± 1.1 | 9.2 ± 1.1 | 0.057 |
| End of study | 9.5 ± 1.1 | 9.4 ± 1.2 | 0.439 |
| Heart Rate (bpm) |  |  |  |
| Screening | 70.6 ± 8.7 | 69.3 ± 9.5 | 0.453 |
| End of study | 71.3 ± 9.0 | 71.6 ± 10.0 | 0.887 |
| Weight (kg) |  |  |  |
| Screening | 73.2 ± 13.1 | 72.5 ± 12.5 | 0.770 |
| End of study | 74.1 ± 13.4 | 72.4 ± 12.5 | 0.509 |
| BMI (kg/m^2^) |  |  |  |
| Screening | 26.7 ± 4.3 | 26.2 ± 3.7 | 0.489 |
| End of study | 27.1 ± 4.3 | 26.2 ± 3.7 | 0.275 |

Abbreviations: BMI, body mass index; bpm, beats per minute; SD, standard deviation.

^†^Between-group comparison, independent Student’s *t*-test.

**Supplemental Table 8.** Summary of adverse events (AEs) over entire study period (n)

|  | Probiotic (N = 50) | | Placebo (N = 50) | |
| --- | --- | --- | --- | --- |
|  | AEs | AEs possibly related to product | AEs | AEs possibly related to product |
| Gastrointestinal disorders | 6 | 3 | 10 | 4 |
| General disorders and  administration site conditions | 1 | 0 | 1 | 0 |
| Infections and infestations | 1 | 0 | 0 | 0 |
| Musculoskeletal and  connective tissue disorders | 1 | 0 | 0 | 0 |
| Nervous system disorders | 0 | 0 | 2 | 1 |
| Skin and subcutaneous  tissue disorders | 1 | 1 | 1 | 0 |
| Vascular disorders | 1 | 0 | 0 | 0 |
